# Bioactive Flavone-*C*-Glycosides of the African Medicinal Plant *Biophytum umbraculum*

**DOI:** 10.3390/molecules180910312

**Published:** 2013-08-26

**Authors:** Anh Thu Pham, Celine Nguyen, Karl Egil Malterud, Drissa Diallo, Helle Wangensteen

**Affiliations:** 1 Section of Pharmaceutical Chemistry, School of Pharmacy, University of Oslo, P.O. Box 1068 Blindern, N-0316 Oslo, Norway; E-Mails: nguyen.celine@gmail.com (C.N.); k.e.malterud@farmasi.uio.no (K.E.M.); 2 Department of Traditional Medicine, Institut National de Recherche en Santé Publique, BP 1746, Bamako, Mali; E-Mail: dri.diallo@yahoo.fr

**Keywords:** *Biophytum umbraculum*, flavone-*C*-glycosides, DPPH, xanthine oxidase, 15-lipoxygenase

## Abstract

Three flavone-*C*-glycosides—cassiaoccidentalin A (**1**), isovitexin (**2**) and isoorientin (**3**)—were isolated from the ethyl acetate (EtOAc) soluble fraction of the methanol crude extract of the African medicinal plant *Biophytum umbraculum*, This is the first report of these compounds in this plant. All compounds were identified by spectroscopic analysis and comparison with published data. Isoorientin (**3**) and the EtOAc extract showed the greatest antioxidant activity in the DPPH assay as well as the strongest inhibition of xanthine oxidase (XO) and 15-lipoxygenase (15-LO). From these results, the extract of *B. umbraculum* might be a valuable source of flavone *C*-glycosides.

## 1. Introduction

*Biophytum umbraculum* Welw. (common syn. *Biophytum petersianum* Klotzsch) (Oxalidaceae) is a slender annual herb distributed in tropical and subtropical Africa, and across to Asia and New Guinea. The aerial parts of the plant have several medicinal uses in Mali and other African countries [1]. Ethnopharmacological surveys on the use of *B. umbraculum* by practitioners in different districts in Mali (Bamako, Siby and Dioila) show that the plant is frequently used against cerebral malaria, but also against hemorrhoids, colonic ailments, wounds, stomach ache and fever [2,3,4]. In Nigeria the plant has been used against wounds, gonorrhea, urethral stones and stomach ache [1]. Other indications reported are constipation, hypertension, migraine, epilepsy, breathing difficulties and lack of fertility [1,5,6]. Various *in vitro* studies indicate that extracts of *B. umbraculum* may exert beneficial pharmaceutical effects on hypertension [5,7,8,9,10]. In addition, pectic polysaccharides isolated from *B. umbraculum* have demonstrated diverse effects on the immune system by virtue of complement fixation [3,4], activation of macrophages and dendritic cells [11], and modulation of intestinal Peyer’s patch cells [12]. Phytochemical investigations on *B. umbraculum* have so far been restricted to polysaccharides [3,4,11,12], although saponins of unknown structure have been stated to be present in aqueous extracts of the plant [13].

In the past decades, there has been a growing interest in antioxidants and free radical scavengers as they may have an important role in the prevention of pathologies in which reactive oxygen species (ROS) or free radicals are implicated, such as atherosclerosis, cardiovascular diseases (CVD), ischemia/reperfusion injury, neurodegenerative diseases and cancer [14,15]. As a part of an ongoing project on Malian medicinal plants [16,17,18], in the present study chemical characterization and investigation of antioxidant activity were performed on *B. umbraculum*.

## 2. Results and Discussion

### 2.1. Isolation and Structural Elucidation

Compounds **1**, **2** and **3** were isolated from the EtOAc soluble fraction of the MeOH crude extract of *Biophytum umbraculum* by column chromatography (CC). Based on the weights of purified fractions, estimated concentrations of substances in the crude MeOH extract are 0.45% (**1**), 0.37% (**2**) and 0.17% (**3**). The compounds were identified as cassiaoccidentalin A (**1**), isovitexin (**2**) and isoorientin (**3**), respectively, by comparing their ^1^H-NMR and ^13^C-NMR spectroscopic data with those reported in the literature [19,20,21]. Besides, the COSY spectrum revealed ^1^H-^1^H couplings and helped assign proton resonances, especially those of the inner sugar of **1**. The HSQC spectrum showed all ^1^J direct ^1^H-^13^C correlations and thus confirmed the assignments of all signals arising from the CH- and CH_2_- groups. Among all the ^1^H-^13^C long-range correlations observed in the HMBC spectrum, the most important were the correlations from the anomeric proton of the inner sugar (H-1′) to C-6 (^2^J), C-5 (^3^J) and C-7 (^3^J) of the aglycone, which confirmed the *C*-glycosylation at C-6.

**Figure 1:**
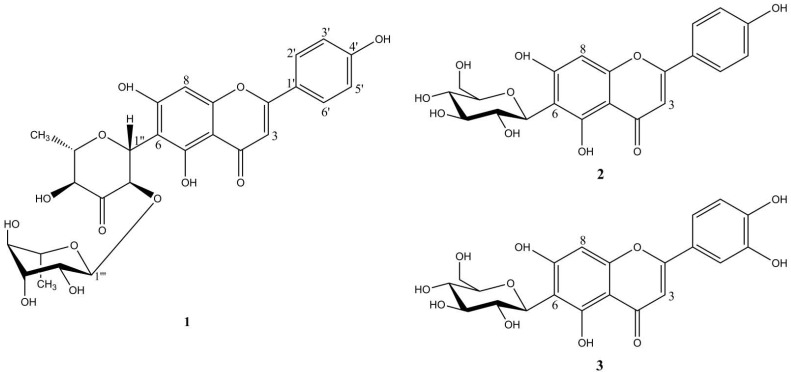
Chemical structures of the three flavone-*C*-glycosides **1**–**3**.

The chemical structures of the three flavone-*C*-glycosides are shown in Figure 1. To our knowledge, this is the first identification of flavone-*C*-glycosides in *B. umbraculum*. Compound **1** is a very rare flavone-*C*-glycoside which has only been identified once before, in *Cassia occidentalis* [19], whereas the more common flavone glucosides **2** and **3** have been identified in another *Biophytum* species, namely *Biophytum sensitivum* [22].

### 2.2. Antioxidant Activity

The dichloromethane (DCM) crude extract of *B. umbraculum* was virtually inactive as a DPPH radical scavenger, xanthine oxidase (XO) and 15-lipoxygenase (15-LO) inhibitor (IC_50_ > 167 µg/mL). The MeOH crude extract and the semi-polar extracts obtained by partitioning the MeOH extract showed fairly high radical scavenger activity and 15-LO inhibition (Table 1). The extracts showed moderate or low inhibitory activity towards XO compared to the positive control quercetin. The EtOAc extract exhibited the highest activity in all three assays.

**Table 1:** DPPH radical scavenging, xanthine oxidase (XO) inhibition and 15-lipoxygenase (15-LO) inhibition of *Biophytum umbraculum* extracts. IC_50_ values ± SD (in µg/mL) are shown.

Extract	DPPH	XO	15-LO
DCM crude extract	>167	>167	>167
MeOH crude extract	13.4 ± 0.6	59.7 ± 6.1	68.9 ± 5.0
EtOAc extract	6.8 ± 0.6	*ca.* 21	43.0 ± 3.6
BuOH extract	12.5 ± 1.9	102.6 ± 5.6	53.4 ± 2.1
Aqueous residue	29.8 ± 3.5	>167	>167
Quercetin (positive control)	4.4 ± 0.4	2.33 ± 0.09	33.4 ± 0.3

The activity of the isolated compounds as DPPH scavengers, XO- and 15-LO inhibitors is shown in Table 2. Flavone-*C*-glycosides were found to contribute both as DPPH scavengers and 15-LO inhibitors in the EtOAc extract. This is in good accordance with previous structure-activity studies indicating the importance of a 2,3 double bond in conjugation with a 4-oxo function in the C-ring and *o*-dihydroxy structure in the B-ring [23,24]. The strongest inhibitor of XO and 15-LO and the best DPPH scavenger was isoorientin. The strong DPPH radical scavenging activity of isoorientin and the much weaker activity of isovitexin are supported by previous results [25,26]. This antioxidant activity clearly shows that the 3′,4′-dihydroxy structural element of isoorientin is of importance for activity, isoorientin having an IC_50_ value similar to the positive control quercetin. This is in accordance with a previous report [27]. The EtOAc extract may contain additional unidentified XO inhibitors, since XO inhibition of the isolated compounds does not account for the activity observed in the EtOAc extract. It has previously been suggested that flavones without a glycosyl group were relatively strong inhibitors of XO, as the presence of a glycosyl group would decrease the inhibitory activity [28,29]. XO and 15-LO inhibitors may be beneficial for diseases and conditions such as ischemia/reperfusion, gout, renal stones, inflammation, arteriosclerosis, neurodegenerative diseases, cancer, aging, *etc.* [28,30,31,32]. Additionally, XO-inhibitors may have beneficial effects as adjuncts in the management of severe *Plasmodium falciparum* malaria [33].

Since the solubility in water of the flavonoids reported here is unknown (although a calculation, as given on the SciFinder website, gives theoretical values of between 0.12 and 0.15 mg/mL), an analysis of aqueous extracts of the plant may be relevant for the evaluation of its ethnopharmacological use, and might thus represent a useful continuation of the present work.

These results imply that the extracts from *B. umbraculum* are a rich source of flavone-*C*-glycosides, and that herbal remedies obtained from this plant may have an effect against inflammations or other diseases related to oxidative stress. The results from our study would seem to be in accord with several of the reported ethnopharmacological usages of the plant.

**Table 2:** Effects of isolated compounds from *B. umbraculum* on DPPH radical scavenging, xanthine oxidase (XO) inhibition and 15-lipoxygenase (15-LO) inhibition. IC_50_ values ± SD (in µM) are shown.

Isolated compound	DPPH	XO	15-LO
Cassiaoccidentalin A (**1**)	>167	149.5 ± 7.4	99.9 ± 2.5
Isovitexin (**2**)	96.0 ± 3.6	>167	107.1 ± 2.1
Isoorientin (**3**)	18.1 ± 1.1	117.2 ± 13.5	86.4 ± 0.5
Quercetin (positive control)	13.7 ± 1.3	7.7 ± 0.3	110.6 ± 1.0

## 3. Experimental

### 3.1. General

1D and 2D NMR spectra were recorded on Bruker DPX 300 or Bruker AVII 400 instruments with CD_3_OD as solvent and TMS as internal standard. CC was done over Sephadex LH-20 (Pharmacia) or reverse phase (RP) silica (VersaPak C18 cartridges; Supelco). Fractions from CC were combined as indicated by TLC. Foils coated with Si gel RP-18 F_254S_ (Merck) were used for analytical and preparative TLC. In analytical TLC, spots were visualized by UV irradiation (254 and 366 nm) and by spraying with Ce(SO_4_)_2_ (1% in 10% aqueous H_2_SO_4_) followed by heating (100 °C, 10–15 min). For UV/VIS measurements, a Biochrom Libra S32 PC instrument was employed.

### 3.2. Plant Material

Flowering, whole aerial parts of *Biophytum umbraculum* Welw. (Oxalidaceae) were collected in Blendio, Mali. The plant was identified by one of the authors, Prof. Drissa Diallo. A voucher specimen (NO. 2653 DMT) was deposited in the herbarium at the Department of Traditional Medicine (DMT), Bamako, Mali.

### 3.3. Extraction and Isolation

The dried and powdered aerial parts of *B. umbraculum* (305 g) were extracted at RT with DCM (4 × 2.5 L), each time for 24 h, yielding 5.1 g of DCM extract (1.7% yield). The plant residue was then extracted similarly with MeOH (5 × 2.5 L) to yield 17.7 g (5.8%) of MeOH extract. The MeOH extract was suspended in 3 × 100 mL distilled water and successively partitioned with EtOAc (6 × 300 mL) and BuOH (5 × 200 mL). The solvents were removed under vacuum, affording an EtOAc extract (4.6 g), a BuOH extract (5.3 g) and an aqueous residue (5.0 g). The EtOAc (E) extract was chromatographed over Sephadex LH-20 (2.5 × 74 cm) with a gradient of H_2_O/MeOH (25%–100% MeOH) to yield 15 subfractions (E1-E15). E9 (334 mg), E10 (1,029 mg) and E11 (555 mg) were chosen for isolation of bioactive compounds based on fraction weights, pattern in NMR spectra and results from activity assays. E9 was flash chromatographed over RP C-18 Si gel (VersaPak, 2.3 × 11 cm) eluting with a H_2_O-MeOH gradient (40%–100% MeOH) to give **1** (57 mg). E10 was applied to a RP C-18 Si gel VersaPak column (4 × 15 cm) and eluted with a gradient of H_2_O-MeOH (20%–100% MeOH) to afford E10.1-E10.9. E10.6 (42 mg) and E10.7 (24 mg) were combined and rechromatographed over a smaller VersaPak column (2.3 × 11 cm) with a H_2_O-MeOH gradient (20%–100% MeOH) followed by preparative TLC to give **2** (8.8 mg). E11 was flash chromatographed with the same column as used for E10 and eluted with a gradient of H_2_O-MeOH (40%–100% MeOH) to yield E11.1-E11.13. E11.2 (50 mg) and E11.3 (21 mg) were combined and purified using the same system to give **3** (6.5 mg). A second batch of plant material (225 g) was subjected to Soxhlet extraction (2 L DCM for 48 h, followed by 3 L MeOH for 48 h). Yield of DCM extract was 7.5 g (3.3%), and of MeOH extract 17.2 g (7.6%). EtOAc extract (4.6 g), BuOH extract (3.3 g) and aqueous residue (5.1 g) were obtained similarly as above.

### 3.4. DPPH Radical Scavenging

Pure substances and crude extracts (Soxhlet) were dissolved in DMSO, and the assay was carried out as reported previously [34]. Quercetin (Sigma-Aldrich) was used as positive control.

### 3.5. Inhibition of Xanthine Oxidase (XO)

Pure substances and crude extracts (Soxhlet) were dissolved in DMSO, and the assay was carried out as reported previously [35]. Quercetin (Sigma-Aldrich) was used as positive control.

### 3.6. Inhibition of 15-Lipoxygenase (15-LO)

Pure substances and crude extracts (Soxhlet) were dissolved in DMSO, and the assay was carried out as reported previously [34]. Quercetin (Sigma-Aldrich) was used as positive control.

### 3.7. Statistical Analysis

All samples were analyzed in triplicates and the results are shown as mean ± standard deviation (SD).

## 4. Conclusions

Our phytochemical study led to the isolation and characterization of three flavone-*C*-glycosides from the aerial parts of *B. umbraculum*. This is the first report on this type of compound from this plant. Compound **3** and the EtOAc extract of the plant revealed strong antioxidant activity towards DPPH radical and 15-LO, and moderate activity towards XO. Further studies on these extracts with respect to antioxidant properties *in vivo* are needed.
